# TLR2 Activation Enhances Inflammatory Signaling and Accelerates Cyst Growth and Renal Fibrosis in Polycystic Kidney Disease Mice

**DOI:** 10.3390/ijms27093853

**Published:** 2026-04-26

**Authors:** Aiping Wei, Yang Zhang, Yu Xu, Jaden Schneider, Nicholas Detterman, Xiaoyun Shu, Tyler Gregersen, Maximo Nahas Folch, Yong Li, Shibiao Chen, Yan Zhang

**Affiliations:** 1 Department of Anesthesiology, The First Affiliated Hospital, Jiangxi Medical College, Nanchang University, Nanchang 330006, China; weiaiping@email.ncu.edu.cn (A.W.); ndyfy05047@ncu.edu.cn (Y.Z.);; 2 Department of Biological Science, College of Sciences and Arts, Michigan Technological University, Houghton, MI 49931, USA; jadensch@mtu.edu (J.S.); mlnahasf@mtu.edu (M.N.F.)

**Keywords:** innate immunity, DAMPs, NF-κB, macrophage

## Abstract

Polycystic kidney disease (PKD), the most common inherited kidney disorder, is characterized by progressive cyst growth and eventual organ failure. Although aberrant innate immune activation is a recognized contributor to PKD progression, the underlying molecular mechanisms remain incompletely defined. Here, we showed that *Pkd1* deletion increased *TLR2* and *MyD88* mRNA expression in renal epithelial cells, indicating enhanced innate immune priming. In vivo, administration of Pam3CSK4 (PAM), a synthetic TLR2 agonist, preferentially amplified pro-inflammatory and pro-fibrotic responses in *Pkd1^RC/RC^* mice compared with wild-type controls, despite inducing similar signaling responses in vitro. Acute PAM treatment for one week rapidly enhanced NF-κB activation in cyst-lining epithelial cells, increased renal inflammation and cell proliferation, and was associated with activation of mTOR signaling and upregulation of c-Myc and Wnt proteins. Sustained PAM treatment further accelerated cyst expansion and renal fibrosis in PKD mice. Importantly, the endogenous TLR2 ligands decorin and biglycan were markedly elevated in human PKD kidneys, supporting the translational relevance of enhanced TLR2 signaling in disease progression. Together, these findings suggest that TLR2 signaling is an important contributor to PKD progression and a potential therapeutic target.

## 1. Introduction

Polycystic kidney disease (PKD), caused primarily by mutations in *PKD1* or *PKD2* genes, is the most common inherited kidney disorder, affecting approximately 1 in 400–1000 individuals [1]. The disease is characterized by abnormal renal epithelial proliferation and transepithelial fluid secretion, leading to progressive formation of fluid-filled cysts and massive kidney enlargement [2,3]. About 50% of PKD patients progress to end-stage renal disease (ESRD) by midlife, and PKD accounts for up to 10% of patients requiring dialysis or kidney transplantation [4]. Tolvaptan is the only FDA-approved therapy for PKD; however, its usage is greatly limited by adverse effects [5]. Therefore, defining the molecular mechanisms that drive PKD pathogenesis remains essential for developing additional effective therapies.

Kidney inflammation is a hallmark of PKD and critically regulates cyst growth and renal fibrosis, two key determinants of renal function decline. Hyperactivation of NF-κB in cyst-lining epithelial cells has been suggested as a key transcriptional regulator of inflammatory signaling in PKD [6,7,8,9]. Elevated levels of proinflammatory cytokines and chemokines, including IL-1β, IL-6, TNF-α, and MCP-1, were consistently detected in the cyst fluid, urine, and serum from PKD patients [10,11,12]. Extensive macrophage accumulation was found throughout the kidney interstitium and even within the cystic epithelium of human PKD [13]. In PKD mouse models, inflammatory pathways are often activated before overt cyst formation, implicating inflammation as a driver of cyst initiation [7,14,15]. Despite these observations, the mechanisms by which inflammation accelerates PKD progression remain poorly understood.

Toll-like receptors (TLRs) are a major class of pattern-recognition receptors (PRRs) that serve as key sensors of innate immunity. Among them, TLR2 and TLR4 expressed in renal tubular epithelial cells have recently emerged as key surveillance receptors in the kidney [16,17]. In addition to pathogen-associated molecular patterns (PAMPs), TLR2 and TLR4 also detect a broad spectrum of damage-associated molecular patterns (DAMPs) released from injured cells or generated during extracellular matrix (ECM) remodeling [18,19]. These endogenous TLR2 and TLR4 ligands have been implicated in inflammatory responses of various kidney diseases [20,21,22]. Upon ligand engagement, TLR2 and TLR4 activate transforming growth factor-β–activated kinase 1 (TAK1), a central signaling node that integrates proliferative and inflammatory pathways [23,24,25].

Growing evidence suggests that TLR2 and TLR4 activation contribute to PKD progression. Elevated TLR2 and TLR4 expression in peripheral mononuclear cells of PKD patients has been shown to correlate with rapid disease progression [26]. Increased expression of DAMPs (HMGB1), TLR2, TLR4, and associated signaling molecules in PKD mouse kidneys suggests an auto-amplifying loop of necrosis and inflammation [27]. Studies using chemical-induced PKD models also support a causal role for TLR2 and TLR4 activation in promoting cystogenesis [28,29]. Within cystic kidneys, both exogenous PAMPs due to cyst infections and endogenous DAMPs generated by injured tissues and ECM remodeling may persistently activate TLR2 and TLR4. Our recent work demonstrated that TLR2 activation stimulates NF-κB-dependent cytokine and chemokine expression and ERK-dependent proliferation in cultured human PKD cells [30], suggesting dual roles for epithelial TLR2 in promoting inflammation and cyst growth. By contrast, TLR4 activation had limited effects in human PKD cells [30].

In this study, we investigated the in vivo effects of a TLR2 agonist on cyst growth, renal inflammation, macrophage accumulation, and fibrosis in *Pkd1^RC/RC^* mice, a well-established orthologous model of PKD. We further examined the direct effects of TLR2 stimulation on NF-κB and ERK signaling pathways in primary renal epithelial cells isolated from PKD mice and the *Pkd1^RC/−^* cell line. To establish clinical relevance, we assessed the expression of TLR2 and its endogenous ligands, including decorin and biglycan, in human PKD kidneys compared with normal human kidneys. Our findings demonstrated that TLR2 agonist treatment promotes inflammatory and proliferative responses that drive cyst progression and renal fibrosis in PKD, suggesting TLR2 as a potential therapeutic target for slowing disease progression.

## 2. Results

### 2.1. TLR2, TLR4, and MyD88 mRNA Are Increased Following Pkd1 Deletion in Primary Renal Epithelial (PRE) Cells

**Figure 1:**
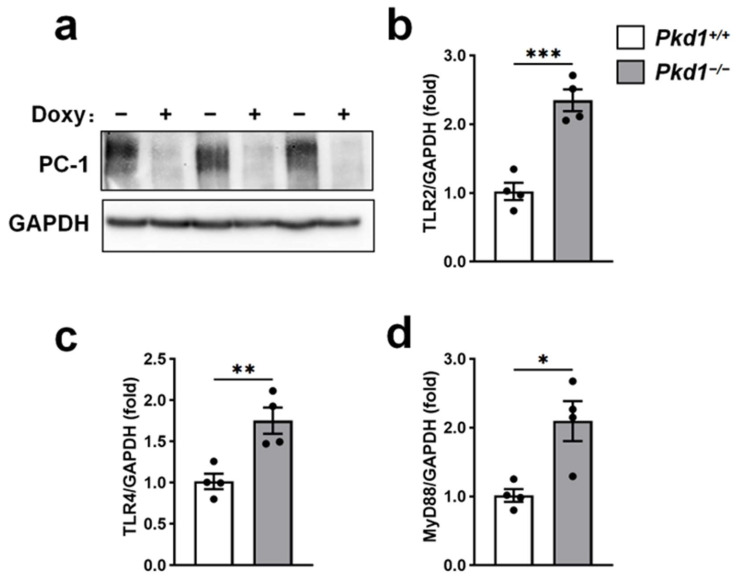
Effect of *Pkd1* deletion on *TLR2*, *TLR4*, and *MyD88* mRNA expression in PRE cells. PRE cells(primary renal epithelial cells, PRE cells) were isolated from 3-week-old *Pkd1^fl/lfl^*; *Pax8-rtTA*; *tetO-7-Cre* mice and cultured in vitro. Doxycycline (1 μg/mL) or vehicle was added to the culture medium for 3 days to induce *Pkd1* deletion. (**a**) Representative immunoblot showing depletion of polycystin-1 (PC-1), encoded by *Pkd1*, following doxycycline treatment. GAPDH was used as a loading control. (**b**–**d**) Relative mRNA expression of *TLR2* (**b**), *TLR4* (**c**), and *MyD88* (**d**) following *Pkd1* deletion. Gene expression was normalized to *GAPDH* and presented relative to the vehicle-treated group (set to 1.0). Data are presented as mean ± SEM (*n* = 4). * *p* < 0.05, ** *p* < 0.01, *** *p* < 0.001.

Our previous studies showed that renal mRNA expression of *TLR2*, *TLR4*, and their adaptor protein *MyD88* was elevated in *Pkd1^RC/RC^* mice, an orthologous PKD model, compared with WT controls [30]. To determine whether these increases are directly attributable to *Pkd1* deletion, we isolated PRE cells from 3-week-old *Pkd1^fl/lfl^*; *Pax8-rtTA*; *tetO-7-Cre* mice. To induce *Pkd1* deletion in vitro, cells were treated with 1 μg/mL doxycycline for 3 days. Western blot analysis confirmed complete depletion of polycystin-1 (PC-1), the protein encoded by *Pkd1*, in doxycycline-treated cells compared with vehicle-treated groups (Figure 1a). Quantitative PCR analysis demonstrated that *Pkd1* deletion resulted in a modest but statistically significant increase in *TLR2* and *TLR4* mRNA expression (2.2-fold and 1.6-fold, respectively; Figure 1b,c). *MyD88* mRNA expression was also elevated by 2.1-fold following *Pkd1* deletion (Figure 1d). These data indicate that loss of functional PC-1 in renal epithelial cells upregulates *TLR2*, *TLR4*, and *MyD88* mRNA expression.

### 2.2. Effects of TLR2 and TLR4 Activation on NF-κB and ERK Signaling Pathways in PRE Cells Isolated from PKD Mice

**Figure 2:**
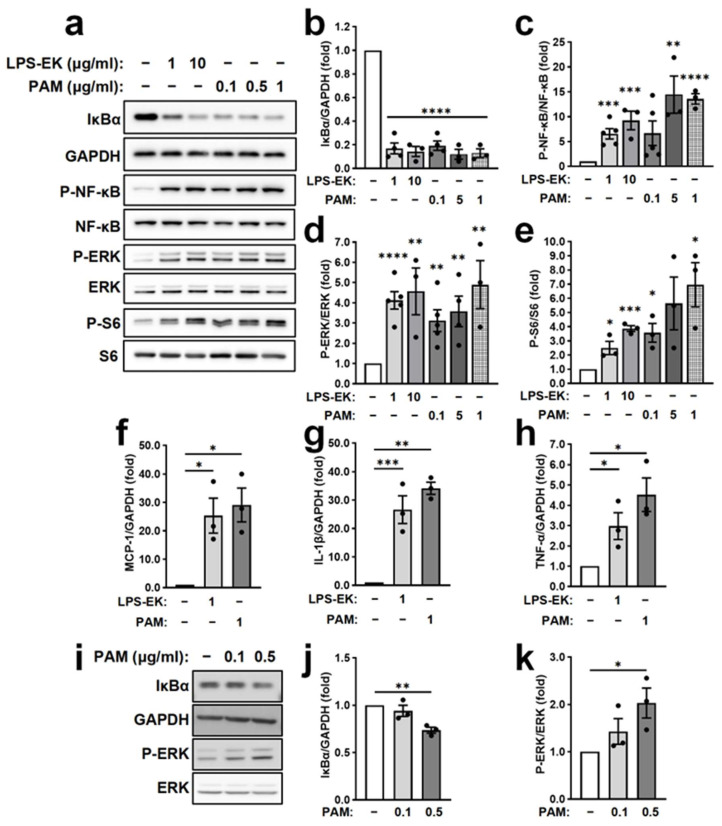
Effects of TLR2 and TLR4 activation on NF-κB and ERK signaling pathways in PRE cells isolated from PKD mice. PRE cells were isolated from 3-week-old *Pkd1^RC/RC^* mice and cultured in vitro. (**a**) Representative immunoblots of PRE cells treated for 30 min with vehicle (culture medium containing 0.1% endotoxin-free H_2_O), ultra-pure LPS-EK (TLR4 agonist), or PAM (TLR2 agonist) at the indicated concentrations for 30 min. GAPDH served as the housekeeping gene. (**b**–**e**) Quantification of IκBα/GAPDH (**b**), P-NF-κB p65/NF-κB p65 (**c**), P–ERK/ERK (**d**), and P–S6/S6 (**e**). Data are presented as mean ± SEM from *n* = 3–5 independent cell preparations, normalized to vehicle (set to 1.0). (**f**–**h**) Relative mRNA expression of *MCP-1* (**f**), *IL-1β* (**g**), and *TNF-α* (**h**) in PRE cells treated with vehicle, 1 μg/mL LPS-EK, or 1 μg/mL PAM for 6 h. Gene expression was normalized to *GAPDH* and presented relative to vehicle-treated WT (set to 1.0). (**i**) Representative immunoblots of *Pkd1^RC/−^* cells treated for 30 min with vehicle (culture medium containing 0.1% endotoxin-free H_2_O) or PAM (TLR2 agonist) at the indicated concentrations for 30 min. GAPDH served as the housekeeping gene. (**j**,**k**) Quantification of IκBα/GAPDH (**j**) and P–ERK/ERK (**k**). Data are presented as mean ± SEM from *n* = 3 independent cell preparations, normalized to vehicle (set to 1.0). * *p* < 0.05, ** *p* < 0.01, *** *p* < 0.001, **** *p* < 0.0001.

Our previous work showed that Pam3CSK4 (PAM, TLR2 agonist) activated NF-κB and ERK signaling in human PKD cells, whereas ultrapure LPS-EK has limited effects [30]. To determine whether similar responses occur in murine PKD cells, we examined PRE cells isolated from *Pkd1^RC/RC^* mouse kidneys, which exhibit elevated expression of TLR2, TLR4, and MyD88 [30]. Immunoblot analysis showed that treatment with PAM or LPS-EK for 30 min dramatically reduced IκBα, an inhibitory protein that sequesters NF-κB in the cytosol, suggesting enhanced NF-κB activation (Figure 2a,b). Treatment with 0.1 μg/mL PAM or 1 μg/mL LPS-EK reduced IκBα levels by approximately 80% (Figure 2b). Both agonists also dose-dependently increased phosphorylation of NF-κB p65 at Ser536 (Figure 2a,c), a critical phosphorylation site for transcriptional activation. At 1 μg/mL, PAM and LPS-EK increased p65 phosphorylation by 13.5- and 6.5-fold, respectively (Figure 2a,c). Consistent with NF-κB activation, qPCR analysis revealed that 1 μg/mL PAM upregulates *MCP-1*, *IL-1β*, and *TNF-α* mRNA by 28.4-, 34.6-, and 4.5-fold, respectively, and LPS-EK exhibited comparable increases in these proinflammatory transcripts (Figure 2f–h).

We next examined proliferative signaling pathways. PAM and LPS-EK treatment for 30 min increased ERK phosphorylation in a dose-dependent manner. At 1 μg/mL, PAM or LPS-EK increased phosphorylated ERK (P-ERK)/ERK ratio by approximately 4.9- and 4.1-fold, respectively (Figure 2a,d). Similarly, phosphorylation of S6, a downstream effector of mTOR signaling, was elevated by 7.0-fold following PAM treatment and 2.5-fold after LPS-EK stimulation (Figure 2a,e).

To determine whether these responses are *Pkd1* mutant-dependent, PRE cells isolated from *Pkd1^+/+^* mouse kidneys were analyzed in parallel. As previously reported [31,32], WT PRE cells respond to PAM and LPS-EK stimulation (Table 1). However, PRE cells from *Pkd1^RC/RC^* mice exhibited significantly greater PAM-induced NF-κB p65 phosphorylation compared with *Pkd1^+/+^* cells (Table 1). In contrast, PAM- and LPS-EK-induced reductions in IκBα and increases in ERK phosphorylation were similar between genotypes (Table 1).

To further determine whether TLR2 activation directly triggers signaling responses in PKD renal epithelial cells, we examined the effects of PAM in *Pkd1^RC/−^* cells, an immortalized PKD renal epithelial cell line. Western blot analysis demonstrated that PAM 0.5 μg/mL stimulation for 30 min decreased IκBα by 30% and increased ERK phosphorylation by 2.0-fold relative to vehicle-treated cells (Figure 2i–k).

Collectively, these data demonstrated that activation of TLR2 or TLR4 enhances NF-κB-dependent inflammatory gene expression and stimulates ERK and mTOR signaling in mouse PRE cells, with augmented NF-κB activation observed in *Pkd1*-deficient cells.

### 2.3. TLR2 Agonist Treatment Selectively Amplifies Renal Inflammation in PKD Mice

Our in vitro studies showed that PAM induced stronger NF-κB p65 activation and mTOR signaling (as indicated by increased P-S6), key regulators of cell proliferation and cyst growth, compared to LPS-EK (Figure 2c,e). In addition, our previous study using human PKD cells demonstrated that the TLR2 agonist exhibited more potent effects than the TLR4 agonist [30]. Therefore, we focused on PAM for subsequent in vivo studies. To determine whether TLR2 activation exerts PKD-specific effects, sex-matched *Pkd1^RC/RC^* mice and *Pkd1^RC/+^* WT controls were treated with 1.0 mg/kg PAM or vehicle once weekly from 3 to 6 weeks of age.

**Figure 3:**
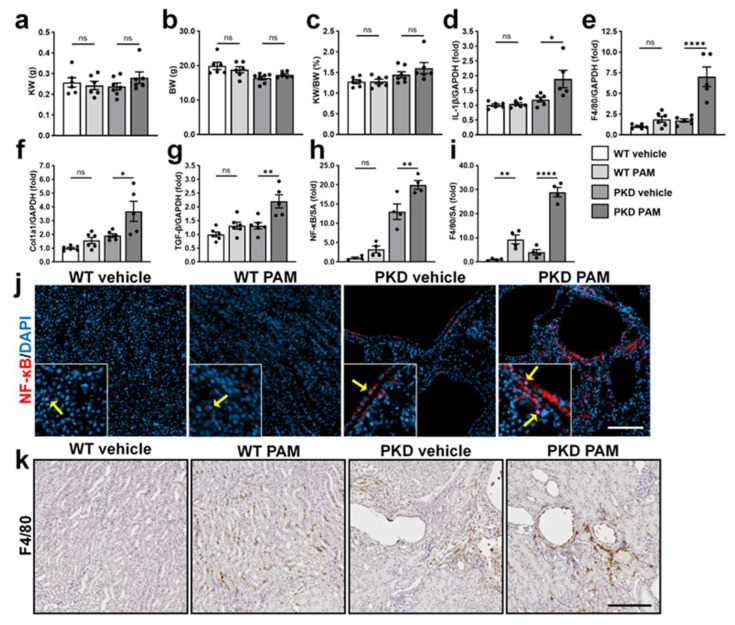
Effects of TLR2 activation on renal inflammatory and fibrotic markers in WT and PKD mice. *Pkd1^RC/RC^* (PKD) mice and *Pkd1^RC/+^* mice (phenotypic WT) were administered sterile saline as vehicle or 1 mg/kg TLR2 agonist Pam3CSK4 (PAM) once a week from 3 to 6 weeks of age. (**a**–**c**) Kidney weight (KW) (**a**), body weight (BW) (**b**), and Kidney weight-to-body weight ratio (KW/BW) (**c**). Data are presented as mean ± SEM (n = 6–7). (**d**–**g**) Renal mRNA expression of *IL-1β* (**d**), *F4/80* (**e**), *Collagen 1a1* (*Col1a1*) (**f**), and *TGF-β* (**g**) in vehicle- or PAM-treated WT and PKD mice. Gene expression was normalized to *GAPDH* and presented relative to vehicle-treated WT (set to 1.0). Data are presented as mean ± SEM. (**j**) Representative immunofluorescence images of kidney sections stained with anti-NF-κB p65 antibody (red) and counterstained with DAPI (blue). Arrows indicate the cellular location of p65 in renal epithelial cells. Scale bar: 200 μm. (**h**) Quantification of NF-κB p65-positive area expressed as a percentage of the total kidney cross-sectional area (SA). (**k**) Representative IHC Images of kidney sections stained for F4/80, a macrophage marker. Scale bars: 100 μm. (**i**) Quantification of F4/80-positive area expressed as a percentage of the total kidney SA. Data are presented as mean ± SEM. * *p* < 0.05, ** *p* < 0.01, **** *p* < 0.0001, ns: not significant.

After 4 weeks of treatment, PAM did not alter the kidney weight, body weight, and kidney weight-to-body weight (KW/BW) ratio in *Pkd1^RC/+^* mice and produced a modest but statistically non-significant increase in *Pkd1^RC/RC^* mice (Figure 3a–c). However, PAM selectively enhanced renal inflammatory and fibrotic gene expression in *Pkd1^RC/RC^* mice compared with WT. Renal *IL-1β* mRNA levels were significantly increased in PAM-treated *Pkd1^RC/RC^* mice, with minimal effect in *Pkd1^RC/+^* controls (Figure 3d). PAM also increased renal *F4/80* mRNA expression by 7.0-fold in *Pkd1^RC/RC^* mice, indicating enhanced macrophage accumulation, whereas effects in WT control kidneys were limited (Figure 3e). Additionally, PAM treatment significantly increased renal TGF-β and collagen 1a1 (Col1a1) mRNA expression by 2.2- and 3.2-fold, respectively, in *Pkd1^RC/RC^* mice, with no significant changes observed in WT controls (Figure 3f,g).

Immunostaining confirmed enhanced NF-κB activation in vivo. PAM increased NF-κB p65 expression, particularly in cyst-lining epithelial cells of *Pkd1^RC/RC^* kidneys, with increased nuclear localization observed following treatment (Figure 3h,j). F4/80 immunostaining further showed increased macrophage infiltration in both genotypes; however, the magnitude of infiltration was greater in *Pkd1^RC/RC^* kidneys (Figure 3i,k). Specifically, PAM treatment increased F4/80 staining by ~10-fold compared with *Pkd1^RC/RC^* mice treated with vehicle (Figure 3i,k).

These findings demonstrated that exogenous treatment with a TLR2 agonist amplifies renal inflammatory and fibrotic responses in *Pkd1^RC/RC^* kidneys to a greater extent than in WT kidneys.

### 2.4. Acute TLR2 Agonist Treatment Enhances Renal Inflammation and Macrophage Accumulation in PKD Mice

**Figure 4:**
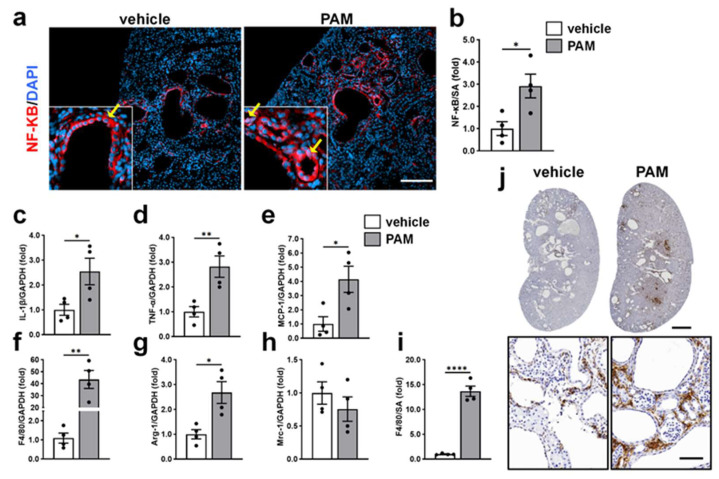
Effects of Acute TLR2 activation on renal tubule NF-κB activation, inflammation, and macrophage accumulation in PKD mice. *Pkd1^RC/RC^* (PKD) mice were administered sterile saline as vehicle or 2.5 mg/kg TLR2 agonist Pam3CSK4 (PAM) on alternating days from 4 to 5 weeks of age. (**a**) Representative immunofluorescence images of kidney sections from PKD mice treated with vehicle or PAM. Sections were stained with anti-NF-κB p65 antibody (red) and counterstained with DAPI (blue). Arrows highlight the cellular location of p65 in renal epithelial cells. Scale bar: 200 μm. (**b**) Quantification of NF-κB p65-positive area expressed as a percentage of the total kidney cross-sectional area (SA). (**c**–**h**) Relative renal mRNA expression of *IL-1β* (**c**), *TNF-α* (**d**), *MCP-1* (**e**), F4/80 (**f**), *Arg-1* (**g**), and *Mrc-1* (**h**) in the indicated groups. Gene expression was normalized to *GAPDH* and presented relative to vehicle (set to 1.0). Data are presented as mean ± SEM (*n* = 4). (**i**) Quantification of F4/80-positive area expressed as a percentage of the total kidney cross-sectional area. Data are presented as mean ± SEM (*n* = 4). (**j**) Representative IHC images of whole-kidney sections from vehicle- and PAM- treated PKD mice stained for F4/80, a macrophage marker. Lower panels show higher-magnification views highlighting macrophage localization within cystic kidneys. Scale bars: 1 mm (upper panels) and 100 μm (lower panels). * *p* < 0.05, ** *p* < 0.01, **** *p* < 0.0001.

Given that TLR2 activation amplifies renal inflammatory and fibrotic responses in *Pkd1^RC/RC^* kidneys to a greater extent than in WT kidneys (Figure 3), we next focus on the temporal dynamics of TLR2-mediated pro-inflammatory effects in *Pkd1^RC/RC^* mice. To induce a robust and rapid TLR2 activation, *Pkd1^RC/RC^* mice were treated with 2.5 mg/kg PAM or vehicle on alternate days for one week. IF staining showed PAM treatment enhanced NF-κB expression, particularly in renal epithelial cells, with enhanced nuclear translocation (Figure 4a,b), consistent with NF-κB activation. Renal mRNA levels of proinflammatory cytokines, the downstream of NF-κB, were significantly increased following PAM treatment. *IL-1β* and *TNF-α* expression increased by 2.5- and 2.8-fold, respectively, compared with vehicle-treated *Pkd1^RC/RC^* mice (Figure 4c,d). *MCP-1* mRNA, a key chemokine mediating monocyte recruitment, was elevated by 4.2-fold after one week of PAM treatment (Figure 4e).

Furthermore, renal mRNA levels of *F4/80*, a macrophage marker, were robustly increased following PAM administration (Figure 4f). To further characterize macrophage subtypes, we assessed Arg-1, a marker associated with pro-proliferative macrophages, and Mrc-1, a marker of resident-derived macrophages. PAM treatment increased *Arg-1* expression by 2.6-fold (Figure 4g) without altering Mrc-1 expression (Figure 4h). Enhanced renal macrophage accumulation was further confirmed by F4/80 immunostaining (Figure 4i,j).

These findings demonstrate that treatment with a TLR2 agonist rapidly induces NF-κB activation, cytokine and chemokine upregulation, and macrophage accumulation in PKD kidneys.

### 2.5. Acute TLR2 Agonist Treatment Increased Cell Proliferation and Activated Signaling Pathways Regulating Cyst Growth in PKD Mice

**Figure 5:**
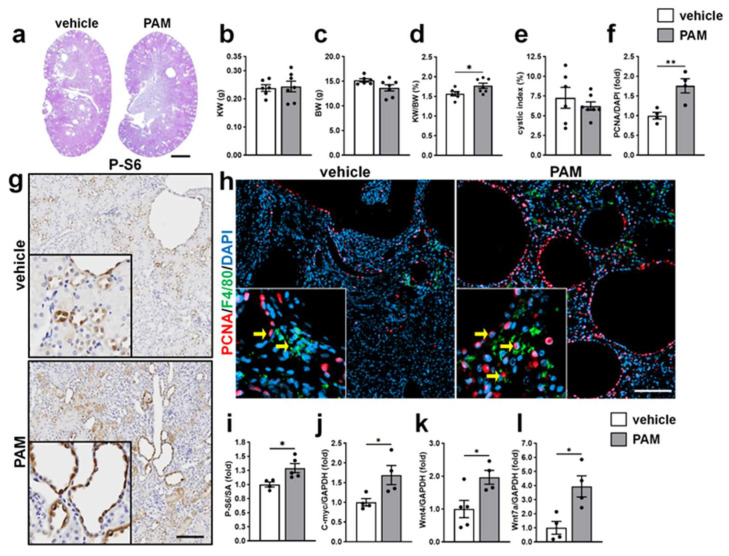
Effects of Acute TLR2 activation on cell proliferation and key signaling pathways regulating cyst growth in PKD mice. *Pkd1^RC/RC^* (PKD) mice were administered sterile saline as vehicle or 2.5 mg/kg PAM on alternating days from 4 to 5 weeks of age. (**a**) Representative H&E-stained kidney sections. Scale bar = 1 mm. (**b**–**e**) Bar graphs represent KW (**b**), BW (**c**), KW/BW (**d**), and cystic index (**e**) calculated as a percentage of cross-sectional surface area (SA). (**f**) Quantification of PCNA-positive nuclei, expressed as a percentage of total nuclei. A minimum of 3 × 10^4^ nuclei were counted per kidney section. (**g**) Representative kidney sections stained for P-S6, a downstream effector of mTOR signaling. Scale bar = 100 μm. (**h**) Representative immunofluorescence images stained for PCNA (red), a proliferation marker, and F4/80, a macrophage marker (green), with nuclei counterstained using DAPI (blue). Arrows highlight the PCNA positive signals in different cell types. Scale bar = 100 μm. (**i**) Quantification of P-S6-positive area relative to total cross-sectional SA. Data are presented as mean ± SEM (*n* = 4–5). (**j**–**l**) Renal mRNA expression of *c-myc* (**j**), *Wnt4* (**k**), and *Wnt7a* (**l**). Data are presented as mean ± SEM. * *p* < 0.05, ** *p* < 0.01.

Our previous work demonstrated that TLR2 activation stimulates ERK-dependent proliferation in cultured human PKD cells [30]. To determine whether TLR2 activation exerts similar proliferative effects in vivo, we examined the impact of acute PAM treatment on cell proliferation and key signaling pathways regulating cyst growth in *Pkd1^RC/RC^* mice. Acute PAM treatment results in a moderate increase in KW/BW (Figure 5d) but does not affect the KW (Figure 5b), largely attributed to a reduction in BW (Figure 5c), and does not alter cystic index (Figure 5e). Despite minimal changes in gross kidney morphology, PAM treatment significantly increased cell proliferation in cystic kidneys. Immunostaining for PCNA, a marker of cell proliferation, revealed a 1.8-fold increase in PCNA-positive cells in PAM-treated *Pkd1^RC/RC^* kidneys compared with vehicle-treated controls (Figure 5f,h). Notably, PAM treatment increased PCNA-positive cells not only in the cyst-lining epithelial cells but also in interstitial cells, including F4/80-positive macrophages (arrows in Figure 5h).

We next investigated signaling pathways known to promote cell proliferation and cyst expansion in PKD, including ERK, mTOR, c-Myc, and Wnt proteins. PAM treatment significantly increased renal phosphorylated S6 (P-S6) levels (Figure 5g,i), indicating activation of mTOR signaling in vivo. In addition, renal mRNA expression of *c-Myc*, *Wnt4*, and *Wnt7a* was significantly elevated following PAM treatment, increasing by 1.7-, 2.0-, and 3.9-fold, respectively (Figure 5j–l). These genes are well-established regulators of epithelial proliferation and cyst growth in PKD. In contrast to our in vitro findings [30], PAM treatment had minimal effects on renal phosphorylated ERK (P-ERK) levels in *Pkd1^RC/RC^* kidneys.

Collectively, these findings demonstrate that acute treatment with a TLR2 agonist enhances cell proliferation in PKD kidneys, associated with activation of mTOR signaling and upregulation of proliferation-associated genes.

### 2.6. Chronic TLR2 Agonist Treatment Accelerated Cyst Growth and Renal Fibrosis in PKD Mice

**Figure 6:**
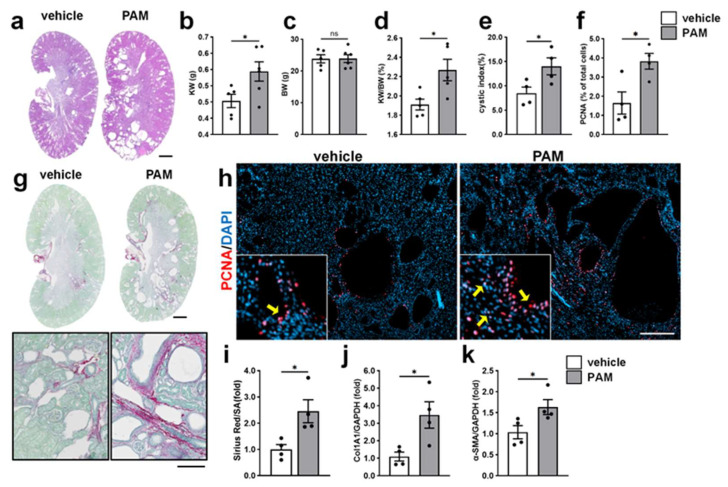
Effects of chronic TLR2 activation on cyst growth and renal fibrosis in PKD mice. *Pkd1^RC/RC^* (PKD) mice were administered sterile saline as a vehicle or 1.0 mg/kg PAM once weekly from 3 to 10 weeks of age. (**a**) Representative H&E-stained kidney sections. Scale bar = 1 mm. (**b**–**e**) Bar graphs showing mean ± SEM (*n* = 5–6) for KW (**b**), BW (**c**), KW/BW (**d**) and cystic index (**e**) calculated as a percentage of cross-sectional surface area (SA). (**g**) Representative images of kidney sections stained with Sirius Red/Fast Green. Sirius Red highlights collagen deposition (red), whereas Fast Green marks non-fibrotic tissue (green). Scale bars = 1 mm (upper panels) and 100 μm (lower panels). (**i**) Quantification of collagen-positive area relative to total cross-sectional SA. Data are presented as mean ± SEM (*n* = 4). (**h**) Representative immunofluorescence images stained for PCNA (red), a proliferation marker, with nuclei counterstained using DAPI (blue). Arrows highlight the PCNA positive signals in interstitial and cyst-lining cells. Scale bar = 200 μm. (**f**) Quantification of PCNA-positive nuclei, expressed as a percentage of total nuclei. A minimum of 3 × 10^4^ nuclei were counted per kidney section. (**j**,**k**) Renal mRNA expression of Collage 1a1 (Col1a1) (**j**) and α-SMA (**k**). Data are presented as mean ± SEM (*n* = 4). * *p* < 0.05, ns: not significant.

*Pkd1^RC/RC^* mice (Balb/C) background showed a rapid renal cyst growth from 3 to 8 weeks of age, followed by a more pronounced pro-fibrotic phase [33]. To assess whether TLR2 activation accelerates both cyst growth and renal fibrosis, 1.0 mg/kg PAM was administered once weekly from 3 to 10 weeks of age to encompass both phases of disease progression. Chronic PAM treatment significantly increased KW, KW/BW, and cyst index (Figure 6b,d,e) compared with vehicle-treated *Pkd1^RC/RC^* mice and did not affect the BW (Figure 6c). Consistently, PCNA staining revealed a significant increase in proliferating cells in kidneys from PAM-treated group (Figure 6f,h), indicating enhanced epithelial cell proliferation. Specifically, the increased PCNA staining was found in both epithelium and interstitial cells, which may be due to increased proliferation of fibroblasts in early fibrosis. In addition, this extended PAM treatment enhanced renal fibrosis. Sirius Red staining demonstrated increased collagen deposition in PAM-treated kidneys (Figure 6g,i). Consistent with these histological findings, renal mRNA expression of fibrosis-associated genes was significantly upregulated, including Col1a1 (3.8-fold) and α-smooth muscle actin (α-SMA; 1.6-fold) (Figure 6j,k). These results demonstrated that sustained treatment with a TLR2 agonist accelerated cyst growth and promoted renal fibrosis in PKD mice.

### 2.7. Expression of TLR2 and TLR4 Endogenous Agonists in Human PKD Kidneys

**Figure 7:**
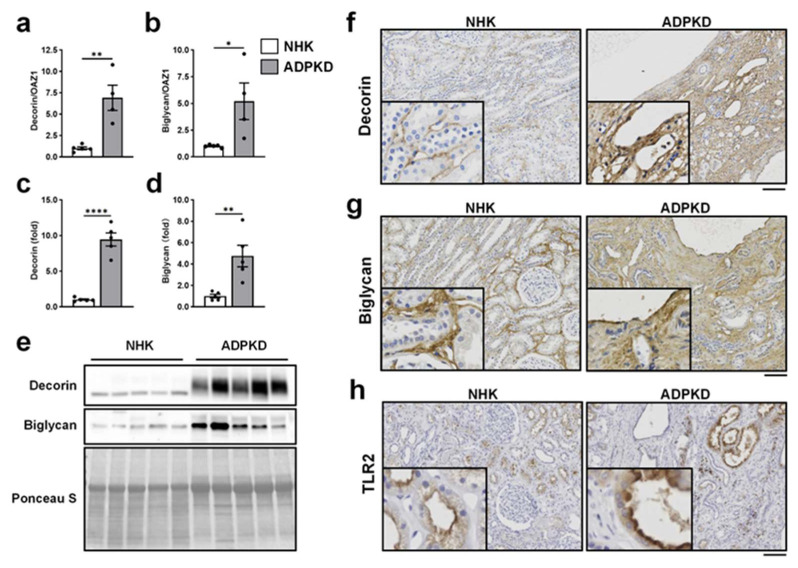
Expression of TLR2 endogenous agonists in human ADPKD kidneys. (**a**,**b**) Relative mRNA expression of *decorin* (**a**) and *biglycan* (**b**) in kidneys from patients with ADPKD and normal human kidneys (NHK). Expression was measured by RT-qPCR, normalized to the housekeeping gene *OAZ1*, and presented as mean ± SEM (*n* = 4). * *p* < 0.05, ** *p* < 0.01. (**c**,**d**) Quantification of decorin (**c**) and biglycan (**d**) core proteins in human ADPKD and NHK tissues following chondroitinase ABC digestion. Protein levels were normalized to Ponceau S staining. Data are presented as mean ± SEM (*n* = 5), normalized to the NHK group (set to 1.0). ** *p* < 0.01, **** *p* < 0.0001. (**e**) Immunoblots showing decorin and biglycan core proteins after chondroitinase ABC digestion. (**f**–**h**) Representative IHC images of kidney sections stained for decorin (**f**), biglycan (**g**), and TLR2 (**h**) in NHK and human ADPKD kidney. Scale bars = 100 μm.

Decorin and biglycan are ECM-derived DAMPs known to activate TLR2 and TLR4 and promote tissue inflammation in various kidney diseases [19,22,34]. Because cystic kidneys are characterized by extensive ECM accumulation, we examined whether these endogenous TLR2 ligands are elevated in human PKD kidneys compared with normal human kidney (NHK). qPCR analysis demonstrated significant upregulation of *decorin* and *biglycan* mRNA in ADPKD kidneys, increased by 6.9- and 5.2-fold, respectively (Figure 7a,b). Consistently, immunoblot analysis showed that decorin and biglycan protein levels were elevated by approximately 9.4-fold and 4.7-fold, respectively, in human PKD kidneys (Figure 7c–e). IHC analysis revealed low expression of decorin and biglycan in NHK, primarily localized to the ECM surrounding renal tubules, which is in line with previous reports [34,35]. In contrast, both DAMPs were markedly increased and accumulated in the interstitium of PKD kidneys (Figure 7f,g). TLR2 was detectable in renal tubular epithelial cells of NHK and showed higher expression in cyst-lining epithelial cells in subsets of PKD kidneys (Figure 7h). These data indicate that increased expression of endogenous TLR2 ligands potentially contributes to human PKD progression.

## 3. Discussion

In this study, we investigated the effects of TLR2 agonist treatment on PKD progression. We demonstrate that *Pkd1* deficiency enhances innate immune priming in renal epithelial cells and sensitizes PKD kidneys to TLR2-mediated activation. Although TLR2 stimulation activates NF-κB and ERK signaling in vitro in both PKD and WT cells, sustained TLR2 agonist treatment in vivo resulted in more pronounced pro-inflammatory and pro-fibrotic responses in *Pkd1^RC/RC^* mice. Acute TLR2 agonist treatment rapidly induces NF-κB-dependent cytokine expression, macrophage accumulation, and epithelial proliferation, whereas chronic activation accelerates cyst expansion and renal fibrosis. Importantly, elevated expression of the endogenous TLR2 ligands decorin and biglycan in human PKD kidneys provides translational support for heightened TLR2 signaling in disease progression. Together, these findings suggested that TLR2 agonists enhanced innate immune responses, cyst growth, and fibrosis in PKD.

TLRs, key PRRs of the innate immune system, respond to both PAMPs and DAMPs to activate inflammatory pathways. In PKD, elevated expression of TLR2 and TLR4 has been linked to disease severity; genetic polymorphisms in these receptors are associated with faster cyst growth and accelerated loss of kidney function in patients [26]. Our previous work demonstrated increased expression of TLR2, TLR4, and their adaptor protein MyD88 in the kidneys of human PKD and *Pdk1^RC/RC^* mice [30]. In the present study, we further showed that *Pkd1* deletion directly upregulates TLR2, TLR4, and MyD88 mRNA expression in primary renal epithelial cells, suggesting that loss of PC-1 intrinsically primes epithelial cells toward heightened innate immune responsiveness. The precise molecular mechanisms linking *Pkd1* deficiency to TLR upregulation warrant further investigation.

Our previous work demonstrated that TLR2 activation stimulated NF-κB-mediated cytokine expression and ERK-dependent proliferation in human PKD cells, whereas TLR4 exerted limited effects. The primary objective of this study was to investigate the in vivo consequences of TLR2 agonist treatment in PKD mice. The *Pkd1^RC/RC^* mouse model exhibits relatively low baseline activation of innate immunity and is therefore well-suited to assess the effects of exogenous TLR stimulation. The present study shows that chronic PAM treatment preferentially amplified inflammatory and fibrotic gene expression in *Pkd1^RC/RC^* mice compared with WT controls, consistent with heightened innate immune priming in *Pkd1*-deficient renal epithelial cells. Acute treatment with PAM for one week rapidly enhanced renal NF-κB activation, cytokine and chemokine expression, and macrophage accumulation, suggesting TLR2 as an upstream regulator of inflammatory amplification in PKD. Renal macrophages are recognized contributors to PKD progression, arising primarily from circulating monocytes and undergoing functional polarization in response to cyst-derived signals [13,36,37]. In this study, PAM treatment markedly increased macrophage accumulation and elevated *Arg-1* expression, a marker associated with pro-proliferative macrophage phenotypes, without altering Mrc-1 expression, which is enriched in resident-derived macrophages. These findings suggest that PAM treatment preferentially promotes recruitment and polarization of monocyte-derived macrophages rather than expansion of resident macrophage populations in PKD kidneys.

Previous studies have identified that TLR2 acts as an upstream regulator of TAK1, which activates both NF-κB and MAPK signaling pathways [24,38]. ERK, a key downstream effector of the MAPK cascade, is an established driver of renal epithelial proliferation and cyst growth in PKD [39,40]. Consistent with our recent findings that TLR2 agonists activate ERK and stimulate human PKD cell proliferation through TAK1 [30], the present study demonstrated that PAM treatment increased ERK and mTOR signaling in PRE cells isolated from *Pkd1^RC/RC^* mice and *Pkd1^RC/−^* cells. In vivo, acute PAM administration enhanced cell proliferation, accompanied by elevated mTOR activation and upregulation of pro-proliferative regulators, including c-Myc, Wnt4, and Wnt7 in *Pkd1^RC/RC^* kidneys. Chronic PAM treatment further accelerated cyst expansion, supporting a sustained pro-proliferative role for this pathway in disease progression. Collectively, these findings indicate that PAM treatment not only amplifies renal inflammation but also promotes epithelial proliferation and cyst growth, likely through coordinated activation of NF-κB–mTOR signaling and downstream proliferative programs.

In addition to promoting proliferation, PAM treatment significantly accelerated renal fibrosis in PKD mice. Progressive interstitial fibrosis is a major determinant of renal functional decline in PKD. Previous studies have shown that overactivation of TGF-β signaling in collecting ducts drives fibrotic remodeling in both wild-type and PKD mice [41], and that epithelial V2R–YAP–CCN2 signaling promotes myofibroblast activation and extracellular matrix deposition in cystic kidney [42]. Although innate immune activation has long been implicated in fibrogenesis in PKD, direct experimental evidence linking specific innate immune receptors to fibrosis progression has been limited. The present study demonstrates that sustained PAM treatment exacerbates fibrotic remodeling in *Pkd1^RC/RC^* kidneys, supporting a mechanistic connection between innate immune activation and renal fibrosis in PKD.

To assess the clinical relevance, we examined the expression of endogenous TLR2 ligands and TLR2 in kidneys from patients with PKD. In addition to recognizing PAMPs, TLR2 responds to a variety of DAMPs. The renal ECM, composed of collagens, proteoglycans, and glycoproteins, represents a major source of DAMPs and undergoes continuous remodeling [19,22]. Proteolytic processing of ECM components can generate bioactive fragments capable of activating PRRs such as TLR2. Decorin and biglycan, two ECM-derived DAMPs, have been implicated in inflammatory and fibrotic kidney diseases [19,22]. Although ECM accumulation is a hallmark of PKD, its expression in human PKD kidneys has not been well characterized. Here, we demonstrate that decorin and biglycan are markedly elevated in the ECM of human ADPKD kidneys compared with normal human kidneys, supporting a potential role for endogenous DAMP-mediated TLR2 activation in disease progression. In addition to DAMP-driven signaling, cyst infection is a common complication in PKD and may increase exposure to microbial PAMPs [43,44]. These PAMPs are potential TLR2 activators and can further exacerbate renal inflammation and promote fibrosis. Such PAMPs are potent activators of TLR2 and could further amplify inflammatory and fibrotic responses within cystic kidneys.

Several discrepancies between our previous and current findings likely reflect species- and cell context-dependent differences in TLR signaling. In human PKD cells, TLR2 activation induced stronger NF-κB activation and ERK-dependent proliferation than in NHK cells, possibly due to elevated MyD88 expression and reduced endogenous MAPK inhibition in PKD epithelium [30,45], while TLR4 activation had limited effects. In contrast, both TLR2 and TLR4 agonists activated NF-κB and ERK signaling in murine PRE cells, suggesting broader responsiveness in the mouse system that may result from species-dependent differences in receptor sensitivity, co-receptor availability, or adaptor recruitment [46]. Notably, TLR2 stimulation preferentially enhanced NF-κB p65 phosphorylation in *Pkd1^RC/RC^* PRE cells compared with *Pkd1^+/+^* controls, whereas IκBα degradation and ERK activation were comparable between genotypes. These findings suggest that *Pkd1^RC/RC^* renal epithelial cells have selectively augmented post-translational activation and transcriptional competence of NF-κB rather than amplifying receptor-proximal signaling events.

Although TLR2 was initially characterized in immune cells such as macrophages, accumulating evidence indicates that renal tubular epithelial TLR2 plays a critical role in kidney innate immunity. TLR2 is predominantly expressed in tubular epithelial cells in the kidney and its expression is upregulated following renal injury [17,32]. Studies using ischemia models and bone marrow chimeric mice have demonstrated that TLR2 expressed in renal parenchymal cells is required for cytokine and chemokine production and contributes substantially to inflammation and tissue injury [17]. In the present study, *Pkd1* deletion directly increased mRNA expression of TLR2, TLR4, and the adaptor protein MyD88 in PRE cells. Moreover, TLR2 and TLR4 agonists activated NF-κB and ERK signaling pathways in PRE cells isolated from *Pkd1^RC/RC^* mice, and enhanced tubular NF-κB activation was observed in vivo following PAM administration. These findings support the hypothesis that TLR2 expressed in tubular epithelial cells contributes, at least in part, to PAM-induced renal inflammation, cyst growth, and renal fibrosis in PKD mice. However, definitive determination of cell type-specific contributions will require future studies employing renal tubule-specific Tlr2 deletion models.

There are several limitations of this study. One methodological limitation of this study is that WT mice were not included in the acute TLR2 activation experiments. Although chronic studies (Figure 3) demonstrate enhanced responsiveness of PKD kidneys to TLR2 activation, WT controls are required to determine whether the acute pro-inflammatory and proliferative responses induced by PAM are specific to the PKD context. The absence of WT mice treated with vehicle or PAM in the acute experiments prevents the assessment of disease specificity. In addition, because PAM was administered via systemic intraperitoneal injection, the relative contributions of kidney-intrinsic versus systemic immune effects cannot be fully distinguished. While PAM may reach the kidney and directly stimulate TLR2 expressed in renal tubular epithelial cells, we cannot exclude the possibility that it also induces systemic inflammation, which may indirectly influence cystic disease progression. Therefore, the observed phenotype likely reflects a combination of systemic and local effects. Future studies using kidney-specific genetic models or localized delivery approaches will be required to delineate the tissue-specific role of TLR2 signaling in PKD. Another limitation of this study is the relatively small sample size (*n* = 3–5) used in some molecular analyses, which may limit statistical power. However, these findings were consistent with phenotypic outcomes, supporting the overall conclusions. In addition, TLR4 agonist induced comparable increases in pro-inflammatory cytokines and chemokines. Although we focused on TLR2 in the current study, investigating the role of TLR4 activation in vivo may provide additional insight and represents an important direction for future studies. Also, Measurement of decorin and biglycan in the PKD mouse model was not performed due to the unavailability of aged *Pkd1^RC/RC^* mice with advanced renal fibrosis. Finally, PRE cells preparations obtained following enzymatic digestion with type IV collagenase consist of heterogeneous renal tubular epithelial populations, including proximal and distal tubular cells. The potential presence of a limited number of fibroblasts and immune cells may influence data interpretation. However, the use of *Pkd1^RC/−^* cells, an immortalized PKD renal epithelial cell line derived from *Pkd1^RC/RC^* mouse kidneys, yielded consistent results with those observed in PRE cells, supporting the robustness of our findings.

## 4. Materials and Methods

### 4.1. Animal Experiments

#### 4.1.1. Mice Information

All mice were housed in the Michigan Technological University (MTU) Animal Care Facility, and all animal procedures were approved by the MTU Institutional Animal Care and Use Committee (IACUC) and conducted in accordance with NIH guidelines. *Pkd1^RC/RC^* (*Pkd1p.R3277C*) mice harbor a knock-in hypomorphic mutation originally identified in ADPKD families and develop a slowly progressive disease phenotype that closely mimics the genetic and pathological features of human ADPKD [47,48]. In contrast, *Pkd1^RC/+^* mice do not develop renal cysts and exhibit normal kidney morphology; therefore, they were used as phenotypic wild-type (WT) controls. *Pkd1^RC/+^* mice were kindly provided by Dr. Darren Wallace at the KUMC with approval from Dr. Peter Harris at Mayo Clinic. *Pkd1^fl/lfl^*; *Pax8-rtTA*; *tetO-7-Cre* mice were provided by PKD-RRC Maryland PKD Research Core.

#### 4.1.2. Mouse Breeding and Genotyping

*Pkd1^RC/+^* mice on the *Balb/C* background were used as breeders to generate both *Pkd1^RC/RC^* and *Pkd1^RC/+^* mice. Genotyping was performed at postnatal days 10–14 (PN10–14) as previously described [30]. Briefly, a 2–3 mm mouse tail was collected for genomic DNA (gDNA) extraction. Samples were incubated in NaOH (50 mM) at 95 °C for 30 min, followed by neutralization with Tris (1M, pH 8.00). PCR amplification was performed using Extract-N-Amp™ PCR ReadyMix™ (E3004, Sigma-Aldrich, St. Louis, MO, USA). PCR products were then resolved on a 1–2% agarose gel, and genotypes were determined based on band size.

#### 4.1.3. TLR2 Activation in *Pkd1^RC/RC^* and WT Mice and Tissue Collection

Pam3CSK4 (PAM, tlrl-pms, InvivoGen, San Diego, CA, USA), a synthetic TLR2 agonist, was used to stimulate TLR2 at doses below those known to induce sepsis. Three complementary in vivo dosing paradigms were used to define genotype dependence, early temporal responses, and long-term disease outcomes of TLR2 activation. The dosing regimens were selected based on commonly used ranges for PAM (TLR2 agonist) in mouse models (1–2.5 mg/kg), which are sufficient to induce inflammatory responses without causing systemic toxicity [49,50]. For the chronic stimulation study and sustained stimulation study, 1.0 mg/kg PAM was administered once weekly to model sustained activation. In contrast, for the acute study, 2.5 mg/kg PAM was administered on alternate days to induce robust and rapid activation of inflammatory and proliferative signaling pathways.

To determine whether the effects of TLR2 activation are PKD-specific, *Pkd1^RC/RC^* and *Pkd1^RC/+^*mice (female and male) received 1 mg/kg PAM or vehicle (sterile saline) via intraperitoneal (i.p.) injection once weekly from 3 to 6 weeks of age. For the acute study, *Pkd1^RC/RC^* mice (both female and male) received 2.5 mg/kg PAM via i.p. injection every other day for one week (from 4 to 5 weeks of age), for a total of 3 injections. For the sustained TLR2 activation study, *Pkd1^RC/RC^* mice (both male and female) received 1.0 mg/kg PAM or vehicle via i.p. injection once weekly from 3 to 10 weeks of age. With *n* = 3–4 male and female mice per group, no apparent sex differences were detected across the three experimental cohorts. A larger sample size would be required to detect subtle differences.

At the end of the treatment period, mice were sacrificed and their kidneys were collected. Kidney-to-body weight (KW/BW) ratios were calculated. Kidneys were bisected longitudinally: one half was fixed in 4% paraformaldehyde (PFA) for histology and immunostaining, and the other half was stored in RNAlater (AM7020, Invitrogen, Waltham, CA, USA) for RNA extraction.

### 4.2. Measurement of the Cystic Index

Tissue sections were stained with hematoxylin and eosin (H&E) staining and imaged using Axio Scan.Z1 (Zeiss, Wixom, MI, USA). Slides were coded, and the total number of cysts, cystic area, and total area of the tissue section were measured by a naïve observer using Image J (version 1.54). Cysts were defined as having a diameter of ≥50 µm, which is 1.8-fold greater than the diameter of the CD.

### 4.3. Immunofluorescence (IF) and Immunohistochemistry (IHC)

Immunostaining was performed as previously described [41,51,52]. Briefly, fixed kidney tissues were embedded in paraffin and sectioned at 5-µm thickness.

For IF, sections were deparaffinized, rehydrated through graded ethanol, subjected to antigen retrieval, blocked, and incubated with the primary antibody overnight at 4 °C. The following day, sections were washed and incubated with appropriate fluorophore-conjugated secondary antibody for 2 h at room temperature. The antibodies used included the following: NF-κB [8242S, Cell Signaling Technology (CST), Danvers, MA, USA], F4/80 (28463-1-AP, ProteinTech, Rosemont, IL, USA), Arg-1 (66129-1-Ig, ProteinTech), proliferating cell nuclear antigen (PCNA; 2586, CST), F(ab’)2-Goat anti-Rabbit IgG (H + L) Cross-Adsorbed Secondary Antibody, Alexa Fluor™ 594 (A-11072, Thermo Fisher Scientific, Waltham, MA, USA), F(ab’)2-Goat anti-Mouse IgG (H + L) Cross-Adsorbed Secondary Antibody, Alexa Fluor™ 594 (A11020, Thermo Fisher Scientific). Slides were mounted using ProLong™ Gold Antifade Mountant with DNA Stain DAPI (P36931, Thermo Fisher Scientific) and scanned with Axio Scan.Z1.

For IHC, deparaffinization, rehydration, and antigen retrieval were performed as described above. Endogenous peroxidase activity was quenched with 3% H_2_O_2_, followed by blocking with normal serum. Sections were incubated with primary antibodies overnight at 4 °C, washed, and incubated with ImmPRESS™ HRP anti-rabbit IgG polymer reagent (MP-7401, Vector Laboratories, Newark, CA, USA) for 30 min at room temperature, and antigen detection was performed using the ImmPACT™ DAB peroxidase substrate kit (SK-4105, Vector Laboratories). Sections were then counterstained with hematoxylin. The following primary antibodies were used: F4/80 (28463-1-AP, ProteinTech), P-S6 (4858, CST), P-ERK (4370, CST), Decorin (14667-1-AP, ProteinTech), and Biglycan (16409-1-AP, ProteinTech). To confirm antigen specificity, rabbit IgG (10500C, Thermo Fisher Scientific) at the same concentrations was used as the negative control. For biglycan staining in human kidney sections, tissues were treated with 7.5 mU/mL chondroitinase ABC (ChABC, C3667, Sigma-Aldrich) for 2 h prior to the blocking step to remove glycosaminoglycan masking and expose the antigen.

### 4.4. Sirius Red Staining

Sirius red staining was performed using the Sirius Red/Fast Green Collagen Staining Kit (9046, Chondrex, Woodinville, WA, USA). Kidney sections were deparaffinized and rehydrated, followed by incubation with the Dye Solution at room temperature for 30 min. After incubation, the Dye Solution was removed, and the sections were rinsed with distilled water, dehydrated through graded ethanol, cleared in xylene, and mounted with a resinous medium. Images were acquired using the Axio Scan.Z1 slide scanner.

### 4.5. Isolation and Culture of Renal Epithelial Cells and Treatment with TLR2 and TLR4 Agonist

Primary renal epithelial cells were isolated from *Pkd1^RC/RC^* mice as previously described [51]. Briefly, 3-week-old mice were euthanized, and kidneys were collected, minced, and digested in 220 U/mL type IV collagenase at 37 °C for 4 h. The digestion was terminated by adding fetal bovine serum (FBS, S11550, R&D Systems, Minneapolis, MN, USA). The cell and tissue pellet was resuspended in DMEM/F12 (SH30023, Cytiva, Marlborough, MA, USA) supplemented with 10% FBS, 0.1% ITS (354351, Corning, Bedford, MA, USA), and Penicillin-Streptomycin (P/S, P0781, Sigma-Aldrich) and cultured overnight to allow cell attachment.

The next day, the medium was replaced with fresh culture medium (DMEM/F12 containing 5% FBS, ITS, and P/S), which was changed every two days until cells reached approximately 80% confluence. Cells were serum-starved in DMEM/F12 supplemented with 0.05% FBS and P/S overnight, followed by treatment with either vehicle (endotoxin-free H_2_O at the same final concentration used for agonist treatments) or increasing concentrations of PAM and ultrapure LPS-EK (TLR4 agonist; tlrl-pcklps, InvivoGen), both dissolved in endotoxin-free H_2_O.

### 4.6. Enzymatic ChABC Deglycosylation Digestion of Human Kidney Lysates

Kidney tissue lysates were extracted in 1% Triton lysis buffer with protease and phosphatase inhibitors as previously described [51]. Chondroitin sulfate chains (CS GAGs) were removed from tissue lysates (20 μg) by chABC enzymatic treatment before performing immunoblot analysis. The samples were digested with 10 mU of chABC (C3667, Sigma-Aldrich) in adequate buffer, Tris-HCl, pH 7.5 (20 mM), and sodium acetate (20 mM) in a final volume of 20 μL overnight at 37 °C as previously described [53]. The deglycosylated protein samples were loaded onto SDS–PAGE and then immunoblotting was performed.

### 4.7. Immunoblot Analysis of Cellular and Tissue Protein Lysates

For cellular experiments, cells were rinsed briefly with cold PBS, and protein was extracted using 1% Triton lysis buffer with protease and phosphatase inhibitors as previous described [30].

Then protein concentration was quantified using Pierce^TM^ Detergent Compatible Bradford Assay (23246, Thermo Fisher Scientific). An equal amount of total protein was loaded onto acrylamide gels, separated by electrophoresis, and transferred to nitrocellulose blotting membrane using the Trans-Blot Turbo Transfer system (1704150, Bio-Rad, Hercules, CA, USA). Membranes were blocked and then incubated with primary antibodies overnight at 4 °C, followed by HPR-conjugated secondary antibodies for detection. The following antibodies were used: PC-1 (E712, Santa Cruz), P-NF-κB p65 (3033T, CST), NF-κB p65 (8242S, CST), IκBα (4814S, CST), P-ERK (4370T, CST), ERK (4695T, CST), P-S6 (4858T, CST), S6 (2217T, CST), Decorin (14667-1-AP, ProteinTech), Biglycan (16409-1-AP, ProteinTech), HRP-conjugated GAPDH (HRP-60004, ProteinTech). Bands were detected with ECL Substrate Kit (SuperSignal^TM^ West Pico PLUS Chemiluminescent or SuperSignal^TM^ West Femto Maximum Sensitivity, Thermo Fisher Scientific), imaged using chemiDoc^TM^ MP Imaging System (12003154, Bio-Rad), and quantified by its analysis software.

### 4.8. RNA Extraction from Tissue and Cells and Quantitative RT-PCR

Total RNA was isolated from kidneys using RNeasy Mini Kit (74104, Qiagen, Ann Arbor, MI, USA). Total RNA from cultured cells was extracted as previously described [30,54]. Briefly, the culture medium was removed, and 0.5 mL TRIzol™ Reagent (15596018, Thermo Fisher Scientific) was added to each well to lyse the cells. Chloroform was added, and after phase separation, the colorless upper aqueous phase was transferred to a fresh tube, followed by the addition of isopropanol to precipitate RNA. After washing with 75% ethanol and air-drying, the pellet was resuspended in RNase-free water. RNA concentration and purity were determined using a NanoDrop spectrophotometer (Thermo Fisher Scientific, Waltham, MA, USA).

First-strand cDNA was synthesized from 2 µg of total RNA using the High-Capacity cDNA Reverse Transcription kit (4368814, Thermo Fisher Scientific). Each PCR reaction contained cDNA templates, 500 nM of forward and reverse primers, and 1 × SYBR Green PCR master mix (4367659, Thermo Fisher Scientific) or SYBR Green qPCR Master Mix (GK10002, Glpbio, Irvine, CA, USA) in a total volume of 20 µL, with reactions run for 40 cycles. Real-time PCR reactions were performed on StepOnePlus^TM^ Real-Time PCR cycler (4376592, Thermo Fisher Scientific) and QuantStudio™ 3 Real-Time PCR System (A28137, Thermo Fisher Scientific). The sequence of primers is listed in Table 2.

### 4.9. Origin and Process of Human Kidney Samples

Kidney sections and tissue of human ADPKD and NHK were provided by the PKD Biomarkers and Biomaterials Core in the Kansas PKD Research and Translational Core Center at KUMC (U54 DK126126), the PKD Research Resource Consortium (PKD RRC). The kidney tissues have been subjected to Enzymatic ChABC deglycosylation digestion followed by immunoblot analysis and IHC analysis as described above.

### 4.10. Pkd1^RC/−^ Cells Culture and Treatment

*Pkd1^RC/−^* cells were obtained from Dr. Veshal Patel at UT Southwestern and cultured in epithelial cell culture medium as previously described [55]. Cells were seeded in 6-well plates with 1.67 × 10^5^ cells per well, the medium was replaced with fresh culture medium (DMEM/F12 containing 2% FBS, Sodium bicarbonate, Sodium selenite, 3,3,5-Triodo-L-Thryonine, insulin, Transferrin, and P/S) and changed every two days until cells reached approximately 80% confluence. The cells were then serum-starved in DMEM/F12 containing 0.05% FBS and P/S overnight, followed by treatment with either vehicle (endotoxin-free H_2_O at the same final concentration used for agonist treatments) or increasing concentrations of PAM dissolved in endotoxin-free H_2_O.

### 4.11. Statistics

Data are presented as means ± standard error of mean (SEM). Statistical significance was determined using an unpaired *t*-test for comparison between two groups. For multiple experimental conditions, we used one-way ANOVA followed by a Dunnett’s multiple comparations test or two-way ANOVA followed by Tukey’s honestly significant difference (HSD) post-test. *p* < 0.05 was taken as significant.

## 5. Conclusions

In conclusion, our study demonstrates that TLR2 agonist treatment accelerated PKD progression. Sustained TLR2 agonist treatment preferably enhanced innate immune activity in *Pkd1^RC/RC^* mice compared with WT mice. Acute TLR2 agonist treatment rapidly promotes NF-κB activation, macrophage accumulation, and epithelial proliferation, whereas sustained activation accelerates cyst expansion and renal fibrosis. The marked elevation of endogenous TLR2 ligands in human PKD kidneys further supports the clinical relevance of heightened TLR2 signaling in disease progression. Collectively, these findings showed that TLR2 agonist treatment enhanced innate immune responses, cyst growth, and renal fibrosis in PKD mice, highlighting TLR2 signaling as a potential therapeutic target.

## Figures and Tables

**Table 1:** Genotype-dependent responses of primary renal epithelial cells to TLR2 and TLR4 activation. Primary renal epithelial cells isolated from *Pkd1^+/+^* and *Pkd1^RC/RC^* mice were treated with PAM, ultrapure LPS-EK (LPS-EK, TLR4 agonist), or vehicle for 30 min. Protein levels of the indicated targets were analyzed by immunoblotting. For each genotype, values were normalized to the respective vehicle-treated group (set to 1.0). Statistical comparisons were performed between *Pkd1^+/+^* and *Pkd1^RC/RC^* cells for agonist-induced changes relative to their respective vehicle controls. * *p* < 0.05, ** *p* < 0.01.

Targets	Treatment (μg/mL)	*Pkd1^+/+^* Cells (Mean ± SEM)	*Pkd1^RC/RC^* Cells (Mean ± SEM)	*p* Values
p-NF-κB/NF-κB	LPS-EK 1	5.17 ± 1.69	6.54 ± 2.32	0.412
	PAM 0.1	2.37 ± 1.59	6.66 ± 5.51	0.248
	PAM 0.5	3 ± 2.31	14.43 ± 6.47	0.045 *
	PAM 1	3.27 ± 3.15	13.57 ± 1.82	0.008 **
IκBα/GAPDH	LPS-EK 1	0.13 ± 0.06	0.17 ± 0.09	0.575
	PAM 0.1	0.5 ± 0.44	0.19 ± 0.08	0.213
	PAM 0.5	0.23 ± 0.15	0.12 ± 0.07	0.310
	PAM 1	0.2 ± 0.1	0.13 ± 0.06	0.362
p-ERK/ERK	LPS-EK 1	4.2 ± 2.31	4.12 ± 0.97	0.946
	PAM 0.1	2.37 ± 0.78	3.12 ± 1.2	0.376
	PAM 0.5	4.03 ± 0.99	3.58 ± 1.51	0.670
	PAM 1	3.73 ± 0.21	4.9 ± 2.05	0.383

**Table 2:** Primer sequence used for RT-qPCR.

	Gene Name	Primer Forward (F) and Reverse (R)
1	mouse-*TNF-α*	F: 5′-ACCCTCACACTCACAAACCAC-3′
		R: 5′-GTGTGGGTGAGGAGCACGTA-3′
2	mouse-*IL-1β*	F: 5′-TGCCACCTTTTGACAGTGATGA-3′
		R: 5′-TGCCTGCCTGAAGCTCTTGT-3′
3	mouse-*MCP-1*	F: 5′-TAAAAACCTGGATCGGAACCAAA-3′
		R: 5′-GCATTAGCTTCAGATTTACGGGT-3′
4	mouse-*F4/80*	F: 5′-TGACTCACCTTGTGGTCCTAA-3′
		R: 5′-CTTCCCAGAATCCAGTCTTTCC-3′
5	mouse-*Arg-1*	F: 5′-CTCCAAGCCAAAGTCCTTAGAG-3′
		R: 5′-GGAGCTGTCATTAGGGACATC-3′
6	mouse-*c-myc*	F: 5′-GATTCCACGGCCTTCTCTCC-3′
		R: 5′-TTCTTGCTCTTCTTCAGAGTCG-3′
7	mouse-*Wnt4*	F: 5′-GCGAGCAATTGGCTGTACCTG-3′
		R: 5′-GCCTTTGAGTTTCTCGCACG-3′
8	mouse-*Wnt7a*	F: 5′-GGAGCTCAAAGTGGGGAGTC-3′
		R: 5′-CTCCTCCAGGATCTTGCTTCTC-3′
9	mouse-*TLR2*	F: 5′-CCTGGGAAGCTAGGGTGAAC-3′
		R: 5′-TCCTCTGAGATTTGACCTCCTTG-3′
10	mouse-*TLR4*	F: 5′-CTCAGCAAAGTCCCTGATGACA-3′
		R: 5′-TCAATTGTTTCAATTTCACACCTGG-3′
11	mouse-*GAPDH*	F: 5′-CCACTCACGGCAAATTCAAC-3′
		R: 5′-GTAGACTCCACGACATACTCA-3′
12	human-*Biglycan*	F: 5′-CGGACACACCGGACAGATAG-3′
		R: 5′-TCCAGGGTGAAGTCCCAGAA-3′
13	human-*HMGB1*	F: 5′-GATAGGGTGGTGTGGAGGAA-3′
		R: 5′-GACATTTTGCCTCTCGGCTTC-3′
14	human-*Decorin*	F: 5′-TCCTTTCCACACCTGCAAACT-3′
		R: 5′-GCCTCTCTGTTGAAACGGTC-3′
15	human-*OAZ1*	F: 5′-CACCATGCCGCTCCTAAG-3′
		R: 5′-GAGGGAGACCCTGGAACTCT-3′

## Data Availability

The data presented in this study are available in the article. Additional data are available from the corresponding author upon reasonable request.

## Abbreviations

The following abbreviations are used in this manuscript:

PKD	Polycystic kidney disease
ESRD	End-stage renal disease
V2R	Vasopressin V2 receptor
AKI	Acute kidney injury
TLRs	Toll-like receptors
PRRs	Pattern-recognition receptors
PAMPs	Pathogen-associated molecular patterns
DAMPs	Damage-associated molecular patterns
ECM	Extracellular matrix
HMGB1	High mobility group box 1
TAK1	Transforming growth factor-β–activated kinase 1
PRE cells	Primary renal epithelial cells
PC-1	Polycystin-1
PAM	Pam3CSK4
WT	Wild type
KW/BW	Kidney weight-to-body weight ratio
PCNA	Proliferating cell nuclear antigen
IHC	Immunohistochemistry
IF	Immunofluorescence
RT-qPCR	Reverse transcription quantitative polymerase chain reaction
NHK	Normal human kidney
ADPKD	Autosomal dominant polycystic kidney disease
